# Exploring physiological stress response evoked by passive translational acceleration in healthy adults: a pilot study utilizing electrodermal activity and heart rate variability measurements

**DOI:** 10.1038/s41598-024-61656-5

**Published:** 2024-05-18

**Authors:** Xiaoru Yu, JiaWei Lu, Wenchao Liu, Zhenbo Cheng, Gang Xiao

**Affiliations:** 1 College of Metrology and Measurement Engineering, China Jiliang University, Hangzhou, Zhejiang China; 2 College of Mechanical and Electrical Engineering, China Jiliang University, Hangzhou, Zhejiang China; 3 Xizi Elevator Co., Ltd., Hangzhou, Zhejiang China; 4 Zhejiang University of Technology, Hangzhou, Zhejiang China

**Keywords:** Translational acceleration, Heart rate variability, Electrodermal activity, Acceleration perception, Stress, Biomedical engineering, Perception, Autonomic nervous system, Stress and resilience

## Abstract

Passive translational acceleration (PTA) has been demonstrated to induce the stress response and regulation of autonomic balance in healthy individuals. Electrodermal activity (EDA) and heart rate variability (HRV) measurements are reliable indicators of the autonomic nervous system (ANS) and can be used to assess stress levels. The objective of this study was to investigate the potential of combining EDA and HRV measurements in assessing the physiological stress response induced by PTA. Fourteen healthy subjects were randomly assigned to two groups of equal size. The experimental group underwent five trials of elevator rides, while the control group received a sham treatment. EDA and HRV indices were obtained via ultra-short-term analysis and compared between the two groups to track changes in the ANS. In addition, the complexity of the EDA time series was compared between the 4 s before and the 2–6 s after the onset of PTA to assess changes in the subjects' stress levels in the experimental group. The results revealed a significant increase in the skin conductance response (SCR) frequency and a decrease in the root mean square of successive differences (RMSSD) and high frequency (HF) components of HRV. In terms of stress assessment, the results showed an increase in the complexity of the EDA time series 2–6 s after the onset of PTA. These results indicate an elevation in sympathetic tone when healthy subjects were exposed to a translational transport scenario. Furthermore, evidence was provided for the ability of EDA complexity to differentiate stress states in individual trials of translational acceleration.

## Introduction

Individuals are frequently exposed to a wide variety of transport scenarios in their daily lives, which are often associated with various forms of passive translational acceleration (PTA). These environmental stimuli can influence psychological stress responses and potentially affect passenger comfort^1,2^. Therefore, it is essential to investigate the patterns of psychological regulation influenced by stress during PTA and to identify relevant biomarkers associated with these responses.

The otolith organs, namely the utricle and the saccule, play a crucial role in the vestibular system as they enable the detection of passive body translations. The experience of stress involves a neurological process that is closely connected to two primary vestibular pathways^3^. The first pathway is associated with the limbic system, specifically the hypothalamus–pituitary–adrenal axis. The second pathway involves the sympathetic component of the autonomic nervous system (ANS). Consequently, PTA has a simultaneous effect on both the individual's stress level and the activation of the ANS^4^.

The term “stress” is used in psychology to describe an emotional state characterized by negative valence and heightened arousal, as outlined in the circumplex model of affect^5^. The psychological processes associated with stress are involuntary, and the autonomic response associated with the presence of a stressor is highly time-dependent^6^. Biosignal characteristics can serve as reliable biomarkers for analyzing such involuntary and time-dependent psychological activity^7^.

The interaction between vestibular input and autonomic nerve activity enables the measurement and assessment of physiological processes mediated by the vestibular system. Electrodermal activity (EDA) serves as a measure of the skin's electrical conductivity, specifically reflecting the activity of sweat glands^8^. The activity of sweat glands is exclusively controlled by the sympathetic nervous system (SNS), making EDA a useful indicator during psychophysiological arousal^9,10^. The skin conductance response (SCR) represents the phasic component of EDA. Previous research has demonstrated an increase in the frequency (SCR_fre_) of SCR in stressful situations^11^. In our analysis, we consider event-related SCR (ER-SCR) information and the complex dynamics of EDA signals, which may provide insights into relevant EDA features in response to an acceleration stimulus on a single trial basis, as well as stress assessment^12^.

It is important to note that skin conductance may not always accurately reflect an individual's subjective experience of stress^13^. The exclusive reliance on EDA data for analysis may result in inconclusive outcomes, underscoring the necessity of integrating multimodal measurements to substantiate the presence of sympathetic dominance. A substantial body of research has been conducted investigating autonomic regulation and stress recognition using EDA and heart rate variability (HRV) signals in a variety of application settings^14,15^. Consequently, we have integrated a combination of EDA and HRV measurements, incorporating both time and frequency domain indices, to fully characterize the physiological stress response^16^.

A randomized controlled trial was conducted to investigate the changes in autonomic nerve activity in healthy adults using measurements of EDA and HRV during exposure to PTA. Subsequently, the extracted EDA and HRV data from the experiments were subjected to an ultra-short-term analysis. In order to assess the stress induced by PTA, an analysis of the complex dynamics of event-related electrodermal activity was conducted at a single trial level.

## Results

A total of fourteen healthy adults (5 females and 9 males) with a mean age of 23.98 ± 4.15(mean ± standard deviation) years met the inclusion criteria and were randomly assigned to the experimental group and the control group. The demographic characteristics of the total sample and each group are presented in Table 1. A comparison of the demographic characteristics between the two groups was conducted, with the following characteristics included: age and sex (female/male). There was no statistically significant difference in terms of demographic characteristics between the two groups. Both groups underwent two stages of physiological data collection: a baseline stage and an intervention stage. In the baseline stage, all subjects stood on a flat surface for a period of 3 min. In the intervention stage, the experimental group underwent five elevator rides, while the control group received a sham treatment. The duration of each trial of the elevator ride in the experimental group lasted approximately 36 s. The intervention stage lasted for 3 min.

**Table 1:** Demographic characteristics (mean ± standard deviation).

	Total sample	Experimental group	Control group	*p*-value
Number of participants	14	7	7	–
Age (years)	23.98 ± 4.15	23.86 ± 3.27	24.29 ± 4.4	ns
Sex (female/male)	5/9	3/4	2/5	ns

The number of participants, the age range of the individuals, as well as the number of females and males in each group and overall, are shown. The results of the chi-square test applied to the characteristics between the two groups are shown in terms of the *p*-value. ns *p*-value ≥ 0.05.

EDA and electrocardiogram (ECG) data were collected and analyzed in both stages of the study. The PTA data was only collected in the intervention stage.

### Physiological effect of PTA via ultra-short-term analysis

The study applied HRV indices, such as the standard deviation of the interbeat interval of normal sinus beats (SDNN), the root mean square of successive differences between normal heartbeats (RMSSD), the low-frequency band power (LF, 0.04–0.15 Hz), the high-frequency band power (HF, 0.15–0.4 Hz) and the ratio of LF-to-HF absolute power (LF/HF). The EDA index employed in the study was the SCR_fre_.

A summary of the comparative and descriptive statistics of HRV and EDA measures for the experimental and control groups in the baseline and intervention stages is presented in Tables 2 and 3. A one-sample *t*-test demonstrated statistically significant differences in the changes of HRV indices RMSSD, HF, and EDA index SCR_fre_ between the two groups. However, no significant differences were found in the SDNN, LF, and LF/HF indices.

**Table 2:** Change-from-baseline values of EDA and HRV indices (mean ± standard deviation) for the experimental and control groups, along with the corresponding *p*-values for the differences between the two groups.

HRV and EDA indices	Experimental group	Control group	*p*-value
ΔSDNN, ms	− 3.118 ± 3.499	− 2.62 ± 3.984	ns
ΔRMSSD, ms	− 4.442 ± 2.385	− 1.042 ± 2.233	*
ΔHF, ms^2^	− 183.970 ± 187.033	− 7.783 ± 36.104	*
ΔLF, ms^2^	− 34.306 ± 117.27	− 96.653 ± 174.193	ns
ΔLF/HF	0.771 ± 1.1	0.15 ± 0.866	ns
ΔSCR_fre_, nSCR/s	0.085 ± 0.056	0.011 ± 0.024	*

Δ change from baseline, ns *p*-value ≥ 0.05, * *p*-value < 0.05.

**Table 3:** EDA and HRV measures (mean ± standard deviation) taken during the baseline and intervention stages for both the experimental and control groups.

HRV and EDA indices	Experimental group			Control group		
	baseline	intervention	*p*-value	baseline	intervention	*p*-value
SDNN, ms	26.508 ± 6.425	22.311 ± 3.964	ns	21.25 ± 5.423	18.757 ± 7.549	ns
RMSSD, ms	24.664 ± 7.717	20.222 ± 6.063	**	17.501 ± 6.372	16.593 ± 8.293	ns
HF, ms^2^	387.388 ± 307.348	200.561 ± 147.107	*	216.333 ± 235.049	209.078 ± 251.273	ns
LF, ms^2^	291.624 ± 75.872	257.319 ± 78.754	ns	248.843 ± 179.915	153.178 ± 115.903	ns
LF/HF	1.555 ± 1.183	2.327 ± 1.675	ns	1.118 ± 0.501	1.268 ± 0.59	ns
SCR_fre_, nSCR/s	0.12 ± 0.034	0.205 ± 0.088	*	0.088 ± 0.048	0.1 ± 0.043	ns

The *p*-values were obtained from the paired samples *t*-test comparing the two stages. ns *p*-value ≥ 0.05, *p-value < 0.05, **p-value < 0.01.

A statistically significant decrease in RMSSD and HF, along with a significant increase in SCR_fre_, was observed within the experimental group in the subsequent within-group analysis using the paired sample *t*-test (see Table 3). The values of RMSSD in the experimental group decreased from 24.664 ± 7.717 ms at baseline to 20.222 ± 6.063 ms in the intervention stage, and from 387.388 ± 307.348 ms^2^ to 200.561 ± 147.107 ms^2^ for HF. The SCR_fre_ increased from a baseline value of 0.12 ± 0.034 nSCR/s to 0.205 ± 0.088 nSCR/s in the intervention stage. However, the other indices of the experimental group as well as all indices of the control group did not show any statistically significant differences between the two stages.

### Analysis of the event-related electrodermal response to PTA

**Figure 1:**
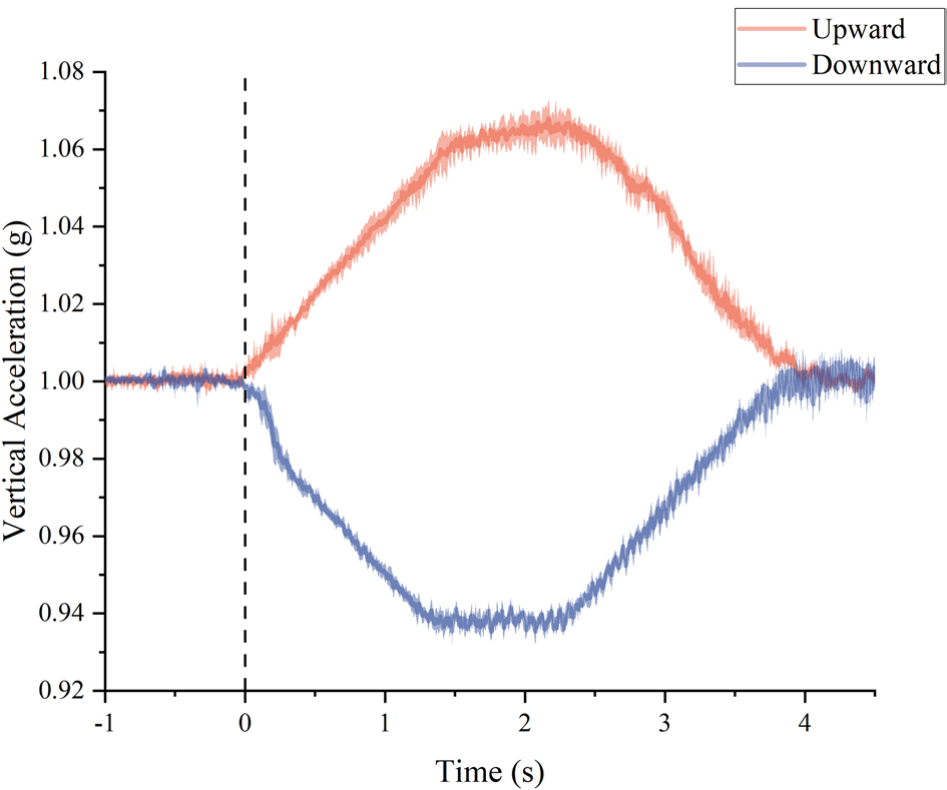
Mean ± standard deviation time series of the vertical upward (red) and downward (blue) acceleration. Data from the elevator over the course of the vertical acceleration are shown. The dashed lines indicate the event time stamp used for event-related electrodermal analysis.

The acceleration process of a single elevator ride is comprised of two phases: acceleration and deceleration. The direction of acceleration was used to categorize each trial of the PTA as either upward or downward (see Fig. 1). The maximum intensity of the upward acceleration was 1.068 ± 0.005 g, while the minimum intensity of the downward acceleration was 0.935 ± 0.002 g. The duration of each PTA trial was determined, with the average upward acceleration lasting 4.055 ± 0.089 s and the average downward acceleration lasting 3.906 ± 0.208 s.

**Figure 2:**
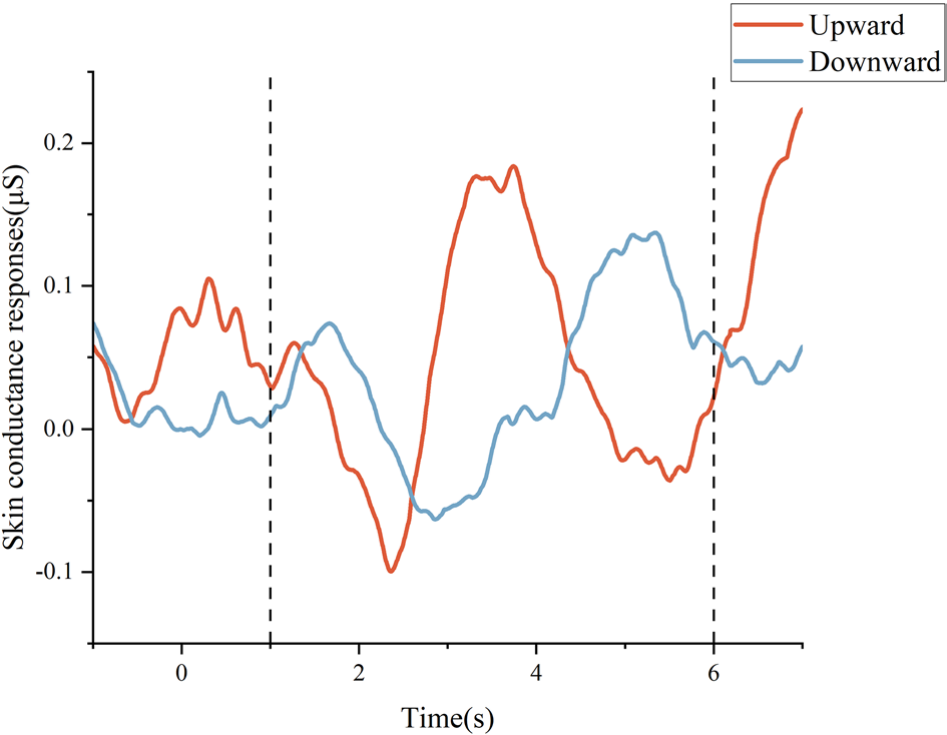
Trial time course of ER-SCR data. The average SCR data was presented from subjects of the experimental group in response to upward (red, n = 32) and downward (blue, n = 34) acceleration. The time interval of 1–6 s following the onset of vertical acceleration was utilized as the SCR time window for subsequent analysis, as indicated by the dashed lines.

The ER-SCR evoked by the upward acceleration was characterized by an amplitude of 0.352 ± 0.574 μS (95% confidence interval[CI] 0.098–0.607 μS), with a latency of 3.328 ± 2.027 s (95% CI 2.429–4.227 s) and a rise time of 0.475 ± 0.369 s (95% CI 0.337–0.612 s). Of the 35 SCR time series related to the vertical upward acceleration events, three did not meet the response criteria, resulting in a 91.4% response rate. For the ER-SCR evoked by the vertical of the downward acceleration, the amplitude was 0.364 ± 0.413 μS (95% CI 0.204–0.524 μS), the latency was 3.461 ± 1.755 s (95% CI 2.779–4.141 s) and the rise time was 0.434 ± 0.410 s (95% CI 0.252–0.615 s). The response rate was 97.1%, as one of the downward acceleration stimulus elicited no electrodermal response. The descriptive statistics of ER-SCR features excluded data from trials without event-related electrodermal responses (see Fig. 2 and Table 4).

**Table 4:** Measures of ER-SCR (mean ± standard deviation and 95% confidence interval) from subjects of the experimental group in response to upward (n = 32) and downward (n = 34) acceleration.

ER-SCR features	Upward acceleration		Downward acceleration	
	Mean ± SD	95% CI	Mean ± SD	95% CI
Latency, s	3.328 ± 2.027	2.429–4.227	3.461 ± 1.755	2.779–4.141
SCR amplitude, μS	0.352 ± 0.574	0.098–0.607	0.364 ± 0.413	0.204–0.524
Rise time, s	0.475 ± 0.369	0.337–0.612	0.434 ± 0.410	0.252–0.615

The latency is the interval between the onset of an acceleration stimulus and the first significant deviation in the SCR signal. The SCR amplitude refers to the magnitude of SCR. The rise time is the time taken from SCR onset to reach peak amplitude within the SCR. SD standard deviation, CI confidence interval.

While it is possible to measure SCR_fre_ to quantify emotional arousal, which represents the strength of a perception, it does not reflect the qualitative aspects of affect or which emotion is present^17^. The complex dynamics of EDA (ComEDA) is an algorithm that demonstrates significant differences in values between stressors and resting states in previous studies^18^. In this study, the EDA data within the event time window will be fed into the ComEDA algorithm for stress assessment, given the highly time-dependent nature of the electrodermal responses.

During a single elevator ride, three intervals of EDA signals were identified in accordance with the onset timing of vertical acceleration and the features of the ER-SCR summarized in the study. The first interval (period I) was established as a baseline, occurring 4 s prior to the onset of vertical acceleration. The second (period II) and third (period III) intervals ranged from 2 to 6 s after the onset of upward and downward acceleration, respectively. A total of 105 intervals were obtained from 35 elevator rides conducted by 7 subjects in the experimental group. ComEDA values were calculated for each interval.

The results of the independent samples *t*-test indicated an increase in period II and period III compared to the baseline period I (see Table 5). The mean ComEDA values of the EDA signals were 0.098 ± 0.052 (mean ± standard deviation, 95% CI 0.079–0.117) for period I, 0.145 ± 0.069 (95% CI 0.121–0.17) for period II, and 0.143 ± 0.056 (95% CI 0.123–0.163) for period III.

**Table 5:** ComEDA values (mean ± standard deviation and 95% confidence interval) of the EDA time series in the three periods, along with the results of *p*-values obtained from the independent samples *t*-test comparing the ComEDA value of period II and period III, respectively, with those of period I.

	Period	mean ± SD	95% CI	*p*-value
ComEDA value	I	0.098 ± 0.052	0.079–0.117	–
	II	0.145 ± 0.069	0.121–0.17	**
	III	0.143 ± 0.056	0.123–0.163	**

SD standard deviation, CI confidence interval, **p-values < 0.01.

## Discussion

The results showed a significant increase in SCR_fre_ and a decrease in RMSSD and HF when subjects were exposed to vertical accelerations during the intervention stage. The findings demonstrated the expected arousal of the sympathetic tone and inhibition of the vagal tone, providing evidence of autonomic response patterns during elevator rides^4^. Furthermore, our study presented a method for determining the duration of accelerated motion. The ComEDA algorithm was applied to the EDA signals obtained from each predefined period, thereby confirming the discriminative power of the complexity of EDA signals between resting states and the intervention of PTA. This methodological approach has the potential to enhance the implementation of physiological measurements in transportation scenarios, with a focus on optimizing the detection of passenger stress in translational transportation scenarios.

The psychophysiological response to PTA is mediated by the extensive projection of the vestibular system to the autonomic network, limbic system, insula, the cingulate gyre, the hippocampus, and the parabrachial nucleus, via cerebellar, brainstem, diencephalic centers, and amygdale cells^19–21^. The main theoretical foundation that has guided our study is the principle that the perception of PTA, as a otolithic input, exerts a direct influence on the autonomic function and stress states^22^.

To achieve a reliable measure of the sympathovagal balance, we combined EDA and HRV measures related to sympathetic and vagal activity. The measurements enabled us to gain insight into the activation of the SNS with inhibition of the vagal nervous system, providing an indicator of our main hypothesis that the acceleration stimuli can result in sympathetic arousal^23^. To elaborate further, a notable elevation in the SCR_fre_ and a reduction in RMSSD and HF power indicates a shift towards sympathetic dominance within the ANS^24^. This indicates the complementary and antagonistic role of the vagal nerve in relation to sympathetic arousal. However, despite some studies showing contradictory results indicating a decrease in SDNN^25,26^, no significant changes were reported during the stimuli in our study. This can be attributed to the fact that RMSSD and HF were more statistically robust and sensitive to heart rate changes in 3-min excerpts compared to SDNN^27^. Furthermore, it's possible that the absence of statistical significance for the SDNN may be attributed to the joint modulation of the index by both branches of the ANS. This analytical interpretation also applies to LF power, which is highly correlated with SDNN.

The onset of the acceleration signals was localized to ensure that the collected EDA data accurately reflected the physiological impact caused by single trial PTA. It should be noted that although we presented the electrodermal responses following the onsets of vertical acceleration, these onset marks did not precisely align with the perceived timing of PTA. Our findings were consistent with the view regarding a vestibular perceptual delay, as they showed an average latency of approximately 3.4 s, which was longer than the typical latency reported in previous studies^28,29^. The limitations of noise caused by spontaneous neuron firing and the perceptual thresholds below which vestibular stimuli cannot be perceived within the human otolith system can be employed to explain this phenomenon^30^. These thresholds are not precise and vary due to personal factors, exhibiting a gradual transition from 0 to 100% probability of detection across a range of values^31^. However, additional research is required to investigate the latency of ER-SCR in response to natural otolith stimuli. The response rate of ER-SCR did not reach 100% in our study. In a previous attempt, we observed even lower response rates of 80% for vertical upward acceleration and 77.5% for downward acceleration. This was achieved by conducting 10 sets of elevator rides with a sample size of 4 subjects. Similar issues arise in the study of response habituation, where the response typically diminishes over a series of stimulus trials^32^. Previous research has demonstrated that vestibular thalamocortical neurons exhibit significantly reduced responses to body orienting movements as a result of contrast gain control adaptation^33,34^. This implies that the influence of vestibular sensory adaptation or habituation may have been crucial in this natural stimulation sequence.

In order to assess the stress induced by vertical acceleration at a single trial level, the EDA signals were segmented into three regions (period I, II and III). These regions correspond to the occurrence of the ER-SCR (ER-SCR latency and rise time). The ComEDA algorithm was then applied. A statistically significant *p*-value was obtained, indicating an elevated ComEDA value within period II and III when compared to the period immediately preceding the acceleration onset (period I). The study conducted by Nardelli et al.^17,18^, demonstrated a significant increase in ComEDA values during mentally stressful tasks. Our findings suggest that our perceptual experiment, which mainly involves the processing and attention to vestibular sensory information, could be considered a potential cause of mental stress. To the best of our knowledge, there has not yet been a study investigating the utility of EDA complexity measurement in response to PTA at the level of individual trials.

Despite the efficacy of our measurements, certain limitations remain. The perception of PTA is a highly interconnected phenomenon within the natural context, involving various sensory systems. Consequently, it is probable that the evoked responses outlined in this paper are not solely attributed to the otolith apparatus but can potentially be influenced by proprioception. The pervasive acknowledgment of this limitation and its extensive discussion in various sources are common knowledge^35^. Another potential limitation of this study lies in the relatively small sample size. A further examination with a larger sample size may be performed for verification. Future studies investigating the potential causes of stress induced by PTA could use neurophysiological measurements to gain further insights into the mechanisms involved in vestibular processing and multisensory integration. Incorporating more sophisticated physiological metrics also helps to develop improved objective measures of acceleration induced stress.

## Methods

### Ethical approval

All subjects gave written informed consent to participate in the study. The study was carried out in compliance with the declaration of the Helsinki Declaration and was approved by the Ethics Committee of China Jiliang University (Grant no. 2023008).

### Subjects

Fourteen healthy subjects (5 females and 9 males) were recruited. Exclusion criteria included any history of neurological or cardiovascular disease, as well as the use of any medications. All subjects were instructed to refrain from consuming caffeine or alcohol, and from experiencing elevator rides on the day of their participation in the experiment. Three additional subjects were excluded from the research sample due to electrodermal non-responding.

### Acceleration data acquisition and analysis

The experiment was conducted in a traction elevator (ca. 5.4 m^2^, 22.9 ± 0.8 ℃ ambient temperature, 64.6 ± 4.7% relative humidity) located in the elevator test tower of Xizi Elevator Co., Ltd., Hangzhou, Zhejiang, China. The elevator has a rated speed of 1.5 m/s and a rated load of 1000 kg. An attitude sensor (WT9011G4K, WitMotion Shenzhen Co., Ltd.) was used to obtain the 3-axis accelerations of the elevator car. The sensor was fixed at the central position of the elevator car platform. The estimation of accelerations (range: ± 16 g, resolution: 0.0005 g/LSB), gyrations (range: ± 4000°/s, resolution: 0.061 (°/s)/LSB), and magnetism (range: ± 2 Gauss, resolution: 0.0667 mGauss/LSB) was achieved through the utilization of a dynamic Kalman filter integrated within the sensor. The signals underwent 16-bit analog-to-digital conversion and were sampled at 1 kHz before being down-sampled to a frequency of 250 Hz. A trigonometric coordinate transformation was then applied to transform the acceleration data into a horizontal-vertical coordinate system using the known gyro data.

### Acceleration event timings

In order to distinguish between the accelerating and non-accelerating states of the elevator, we collected acceleration data while the elevator was stationary for 1 min. After preprocessing the acceleration data, we applied a two-tailed one-sample *t*-test with α = 0.05 to obtain the rejection region. The accelerating state of the elevator was determined by the presence of more than 0.5 s of continuous vertical acceleration data within the rejection region.

A one-sample *t*-test (two-tailed, α = 0.05) was conducted to determine if there was any significant difference in acceleration in the horizontal axis parallel to the ground between the elevator in a stationary state and its moving state. The acceleration of the horizontal axis is the Euclidean norm for both the X-axis and Y-axis. The results showed no significant difference between the two states (*p*-value ≥ 0.05). All the onset and end timestamps of PTA were subject to manual inspection.

### Physiological data acquisition and analysis

The BIOPAC MP150 system was used to record the ECG and EDA at a sampling rate of 1 kHz. Data analysis was performed using Acqnowledge 5.0 software. All electrode placements were made using disposable Ag/AgCl electrodes (EL504, Biopac Systems Inc., Goleta CA, USA) filled with electrode paste.

A 0.5–40 Hz bandpass filter was applied to process the ECG data. The ECG data was then downsampled to 250 Hz. The electrodes were placed for signal recording with the positive electrode on the left upper limb, the negative electrode on the right upper limb, and the ground reference on the right lower limb. The HRV was calculated based on the R-R interval extracted from the ECG signals. The frequency domain analysis of HRV was conducted using Fast Fourier Transformation.

Following the recommendations of Fowles et al.^36^, we measured EDA using two electrodes attached to the distal phalanges of the index and middle fingers on the non-dominant hand. The raw EDA data underwent preprocessing using a lowpass filter with a cut-off frequency of 35 Hz and were then down-sampled to 250 Hz. As a step in the data filtration process, we visually inspected the graphed EDA data to identify and eliminate motion artifacts throughout the entire experiment. The analysis of EDA typically involves two components: the SCR and the skin conductance level. The SCR is the phasic component, which indicates a rapid and smooth increase in dermal activity in response to stimuli. To obtain the SCR signals, the preprocessed EDA data was filtered using a 0.05 Hz highpass filter and a minimum criterion of 0.05 μS for the SCR amplitude^37^. The number of SCR per minute was used to measure the SCR frequency during each stage of the experiment. The ER-SCR was defined as the response occurring within 1–6 s after the onset of each acceleration trial. Furthermore, we categorized the ER-SCR based on the direction of the acceleration.

### Data synchronization and alignment

A virtual button was programmed to send timestamps to both the attitude sensor and BIOPAC system, enabling precise synchronization of the raw acceleration and physiological data streams. The timestamps were automatically sent at one-minute intervals upon activation of the button. This alignment of the two data streams was made possible due to their shared sampling rate. To reduce alignment errors caused by dropped frames, we used MATLAB's 'interpl' function to interpolate each 1-min segment of the raw data to 60,000 time points.

### Experimental protocol

The study involved randomizing all subjects into two equal-sized groups: the experimental group and the control group. Throughout the experiment, environmental noise was minimized using noise-canceling headphones (SONY WH-XB910N).

Both groups participated in two stages of data collection: a baseline stage and an intervention stage. The participants' EDA and ECG data were recorded in both stages. In the intervention stage, in addition to the aforementioned data, the attitude data of the elevator car were also recorded.

**Figure. 3:**
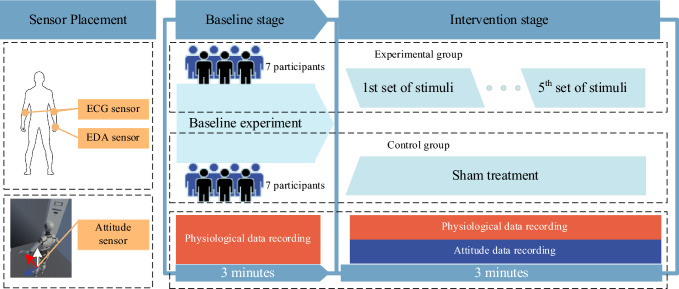
Experimental flowchart. All participants were randomly allocated to two equal-sized groups: the experimental group and the control group. ECG and EDA data were recorded during both the baseline and intervention stages, with each stage comprising a three-minute session. During the intervention stage, participants in the experimental group were exposed to five elevator rides, while participants in the control group received a sham treatment. The attitude sensor, positioned at the center of the elevator, recorded the elevator's attitude data during the intervention stage.

During the baseline stage, all subjects stood on a flat surface with bare feet and closed eyes for 3 min in a resting state. The experimental group differed from the control group in that they were exposed to five elevator rides during the intervention stage, while the control group remained stationary. The experimental elevator rides were initiated irregularly by the recording personnel and completed within approximately 36 s. The intervention stage lasted for 3 min. Figure 3 presents an outline of the experimental procedure.

### Statistical analysis

The study's full set of indices includes SDNN, RMSSD, HF, LF, LF/HF, SCR_fre_, and ComEDA values. All statistical tests were performed using SPSS software (IBM SPSS Statistics, Version 26.0), and results were considered statistically significant at *p* < 0.05. The HRV and GSR indices were found to be normally distributed based on the Kolmogorov–Smirnov test and visual inspection of the normal QQ plot, and all met the normality criteria. An independent samples *t*-test was conducted to examine whether the measured changes of responses evoked by acceleration stimuli in the experimental group, on the aforementioned indices (excluding ComEDA), were equal to those in the control group. The statistically significant indices identified in the independent samples *t*-test will be further compared between the intervention stage and the baseline stage within both groups using the paired samples *t*-test. For the ComEDA value, we conducted an independent samples *t*-test to compare period II and period I, and simultaneously, to compare period III and period I.

## Data Availability

The datasets used and/or analyzed during the current study are available from the corresponding author on reasonable request. Data requests can be made via email (yuxiaoru@cjlu.edu.cn).
